# A Tooth Segmentation Method Based on Multiple Geometric Feature Learning

**DOI:** 10.3390/healthcare10102089

**Published:** 2022-10-20

**Authors:** Tian Ma, Yizhou Yang, Jiechen Zhai, Jiayi Yang, Jiehui Zhang

**Affiliations:** College of Computer Science and Technology, Xi’an University of Science and Technology, Xi’an 710600, China

**Keywords:** tooth segmentation, geometric feature learning, 3D shape segmentation, deep learning

## Abstract

Tooth segmentation is an important aspect of virtual orthodontic systems. In some existing studies using deep learning-based tooth segmentation methods, the feature learning of point coordinate information and normal vector information is not effectively distinguished. This will lead to the feature information of these two methods not producing complementary intermingling. To address this problem, a tooth segmentation method based on multiple geometric feature learning is proposed in this paper. First, the spatial transformation (T-Net) module is used to complete the alignment of dental model mesh features. Second, a multiple geometric feature learning module is designed to encode and enhance the centroid coordinates and normal vectors of each triangular mesh to highlight the differences between geometric features of different meshes. Finally, for local to global fusion features, feature downscaling and channel optimization are accomplished layer by layer using multilayer perceptron (MLP) and efficient channel attention (ECA). The experimental results show that our algorithm achieves better accuracy and efficiency of tooth segmentation and can assist dentists in their treatment work.

## 1. Introduction

Profitting from the rapid development of computer graphics, wireless communication and other technologies, the entire field of dentistry has developed toward digitalization and intelligence. New invisible orthodontic tools have emerged, represented by the virtual orthodontic system. Generating treatment plans through the virtual orthodontic system can reduce patients’ long orthodontic treatment time, ease the pain of the orthodontic process, and reduce patients’ medical costs. The virtual orthodontic system allows the dentist to understand the patient’s oral condition more easily, efficiently and visually, thus assisting the dentist in developing a better treatment plan and allowing the patient to achieve the desired orthodontic results faster and better [1].

The purpose of tooth segmentation is to segment each tooth individually in the 3D orthodontic model. This is a key part of the orthodontic process and the most essential component of the virtual orthodontic system. The accuracy of segmenting the teeth affects the accuracy of the subsequent treatment work (e.g., tooth alignment, simulated root generation). Furthermore, the intelligent and efficient segmentation of the teeth saves the dentist much time and shortens the orthodontic treatment cycle. However, there are many difficulties in segmenting each tooth in the dental model. Specifically, the human dental model is a 3D model with complex appearance, similarity areas and unique shape. The junction between teeth and gums has blurred shape changes, and the tooth boundary is not obvious due to crowded, misaligned or missing teeth. All these difficulties make the tooth segmentation process error prone. Therefore, how to design an accurate and efficient tooth segmentation algorithm is a key problem in orthodontics.

In recent years, point cloud segmentation has become a highly regarded research direction in 3D shape segmentation. Since dental models can be transformed into point cloud models, the study of point cloud segmentation can play a guiding role for tooth segmentation. Since the pioneering work of PointNet [2] set off an upsurge in the field of 3D point cloud segmentation, have explored more accurate and efficient point cloud segmentation methods from the perspectives of multilayer perceptrons (MLPs), convolutional neural networks, graph neural networks, attention mechanisms, and so on. Subsequently, excellent schemes such as PointNet++ [3], GACNet [4], and RandLA-Net [5] emerged. RandLA-Net has achieved impressive performance in the field of cloud segmentation in large scenic spots. This is mainly due to its simple and efficient sampling method and powerful local feature aggregation module. However, the input of the network is usually only point coordinate features, and the corresponding processing flow is not designed for other important geometric features (such as normal vectors). The tooth features to be learned by the network are missing and prone to erroneous segmentation.

In addition to the above ideas, in the field of deep learning methods, researchers have also studied tooth segmentation methods in two directions. One is to first convert the 3D dental model into 2D image data and then use the already more mature 2D CNN to extract dental features and guide the segmentation of 3D teeth [6,7,8]. However, this conversion process is usually accompanied by noise which results in the loss of tooth features. The other approach is to extend the existing point cloud segmentation network so that it can handle the dental mesh model and complete tooth segmentation [9,10]. However, these methods model the coordinates of the mesh vertices in the local region in a simpler way and do not sufficiently take into account the relationship between the normal vectors of the different meshes in the local region. This leads to the inability of the network to learn detailed features, which affects the accuracy of the final tooth segmentation results.

In view of this, this paper extends and improves the existing point cloud segmentation method [2,5] to achieve end-to-end tooth segmentation on a dental mesh model. A multiple geometric feature learning module is designed, which enables the information of geometric features with different attributes to be learned in a complementary way by encoding the enhanced centroid coordinates and normal vectors of each triangular mesh. In addition, for local to global fusion features, MLP and ECA [11] are used to perform feature downscaling and channel optimization in a layer-by-layer cascade manner to complete tooth segmentation accurately and efficiently. The rest of the paper is organized as follows. Section 2 explores related works that segmentation of teeth from 3D dental models using traditional or deep learning techniques. Section 3 outlines our methodology. Section 4 presents and discusses our results. Finally, Section 5 presents the conclusions of this paper and the prospects for future work.

## 2. Related Works

### 2.1. Traditional Tooth Segmentation Methods

Kondo et al. [12] calculated a panoramic image of the tooth model using the dental arch obtained from the depth image as a reference. They then used it to detect the gaps between the teeth and determine their location and orientation, and finally segmented the teeth and gums using the gingival margin line. However, the segmentation result of this method is poor when the tooth model has malformation. Hao et al. [13] proposed an interactive tooth segmentation method. The method uses the vertex curvature value of each triangular mesh and the curvature threshold input by the user to obtain the feature region. The skeleton line of the feature region is obtained by the morphological method, and the segmentation line is obtained by continuous refinement of the skeleton line to finally complete the tooth segmentation. However, this method needs to fill in part of the interrupted part in the feature area manually.

Yuan et al. [14] achieved tooth segmentation by identifying the adhesion areas between teeth and gums and teeth in the 3D dental model, removing the adhesion areas between adjacent teeth, and reconstructing the missing surfaces of teeth. However, identifying the adhesion region link requires many manual operations and consumes the user’s time. Kronfeld et al. [15] first calculated the segmentation line between teeth and gingiva, obtained the arch position of a single tooth and then used the snake method to segment the teeth. However, the segmentation effect was poor in the case of malformation in the dental model. Wu et al. [16] used a morphological skeleton segmentation algorithm and a regional growth method to complete the segmentation of dental mesh models. However, when there is noise in the dental model data, considerable human-computer interaction time is required to ensure the accuracy of segmentation. Zou et al. [17] proposed a weighted harmonic field algorithm to segment teeth, which can perform better on malformed dental models. However, it requires more frequent human–machine interactions, and the segmentation efficiency is low.

In summary, traditional tooth segmentation methods start from the low-level geometric features of the dental model itself (watershed algorithm [18,19], region growth method [16,20], snake algorithm [15], harmonic field method [17]) or explore the auxiliary information from the 2D image of the dental model to solve the 3D model segmentation [12]. These methods usually have disadvantages, such as large segmentation errors in the presence of occlusion and deformation of the dental model, ease of producing oversegmentation, insufficient automation of seed point selection, frequent human–machine interaction; This burdens the user, and leads to large computational efforts.

### 2.2. Tooth Segmentation Methods Based on Deep Learning

Xu et al. [8] proposed a two-stage hierarchical CNN structure for tooth segmentation, where one is used for gingival and tooth labeling and the other for interdental labeling. The CNN model is first trained using 2D tooth feature images, after which the segmentation results are optimized using several steps, such as boundary-aware simplification, fuzzy clustering, and boundary smoothing. However, the 2D tooth feature images are transformed from the simplified tooth model, which will lose the detailed features. Sun et al. [21] proposed an automatic tooth seed point picking method based on FeaStNet [22], which can efficiently mark the seed points of teeth and later combine with the region growing method to complete tooth segmentation. However, manual correction of incorrect seed points is needed. Tian et al. [23] proposed an automatic tooth segmentation method based on hierarchical feature learning. First, the original dental model is preprocessed to obtain the octree model of teeth with labels. Next, the classification recognition among teeth is completed by using the hierarchical feature learning-based method. Finally, the segmentation of single teeth is completed by using 3D CNN and the conditional random field. The segmentation edge of this algorithm is close to the real segmentation result, but the generalization ability of the network needs to be enhanced. Zhang et al. [24] proposed a tooth segmentation algorithm based on the point cloud data of the mouth scan reconstructed from cone beam CT (CBCT), which mainly consists of an instance segmentation network and a fine-grained segmentation network. The former is used to obtain the shape and relative position information of the teeth, and the latter is used to achieve accurate semantic segmentation of a single tooth. However, when the teeth overlap, the tooth segmentation results will have errors. Wang et al. [25] borrowed the network structure design of U-Net [26] and used the KPConv [27] convolutional kernel to construct a 3D tooth segmentation neural network. It has good segmentation results for molar teeth, but the segmentation effect of other parts of teeth is yet to be verified.

Lian et al. [8] proposed an end-to-end deep neural network that first learns multiscale contextual features and overall features from a dental mesh model. it then integrates these features using a dense fusion strategy to finalize tooth segmentation. However, this algorithm computes two adjacency matrices at different scales, which leads to long training time and high memory consumption. Zanjani et al. [28] borrowed the idea of PointCNN [29] and proposed a neural network that directly utilizes the original dental model without downsampling to achieve accurate tooth segmentation. However, this network requires a large amount of data and consumes a significant amount of memory resources at runtime. This imposes some limitations for implementation. Cui et al. [30] proposed a two-stage neural network, which first detects all the teeth by the tooth center of mass and then segments each detected tooth. Zhang et al. [31] proposed a dual-flow graph convolutional neural network, which separately processes the vertex and normal vector features of the dental mesh model and then fuses the two obtained geometric features to obtain the tooth segmentation results.

In summary, deep learning-based segmentation methods usually have higher segmentation accuracy and require less human-computer interaction than traditional methods. This is mainly due to the extraction and use of higher-level geometric features and the fusion of features at different levels by neural networks. In addition, the user does not need to provide a priori knowledge (e.g., manual seed point labeling) for the segmentation process. However, since the vertex coordinates and normal vectors represent the geometric features of each triangular mesh from different perspectives, if the processing of vertex coordinates and normal vectors is not effectively distinguished, this can lead to a failure to produce a complementary interplay of feature information between the two. Therefore, the algorithm in this paper encodes the enhancement of these different geometric information and performs sufficient feature learning to improve the accuracy of tooth segmentation.

## 3. Method

In this paper, we propose a tooth segmentation method based on multiple geometric feature learning, which consists of the following two main parts.

**Figure 1:**
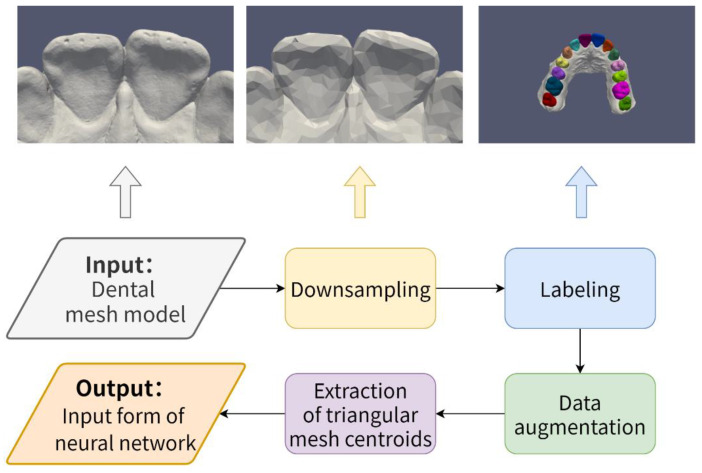
Creation and preprocessing of dental model datasets.

(1) Creation and preprocessing of dental model datasets. As shown in Figure 1, it includes downsampling of the dental model, labeling of the dental model, data augmentation, and extraction of each triangular mesh centroids;
(2) Construction of a tooth segmentation network based on multiple geometric feature learning.

### 3.1. Creation and Preprocessing of Dental Model Datasets

The data used in this paper are original 3D dental models of 32 cases acquired by an intraoral scanner and saved on a computer system in the file format STL (STereoLithograph). Dental models with incomplete or severely malformed scan reconstruction results are removed. The original dental model contains approximately 350,000 triangular meshes, which is limited by training resources and generally requires a downsampling operation to reduce the model size while trying to retain the key shape structures of the original dental model. Therefore, referring to some previous works [10,21,25], the number of triangular meshes is reduced to 10,000 in this paper. As shown in Figure 1, on the downsampled dental models, we follow the recommendations of professional dentists and label each dental model into 15 different semantic parts, including the gingiva as well as each incisor, canine, premolar, and molar. Normal teeth in the data set account for 70% of the data and teeth with malformations account for 30% of the data (e.g., crowded teeth, scattered gaps, etc.). In the testing section, we used 56 teeth from 4 different dental models to complete the test. To further expand the training dataset, the following data augmentation methods were applied to the annotated dental models in this paper:

- Random translation. Along any coordinate axis in three-dimensional space, the dental model undergoes a small translation;
- Construction of a tooth segmentation network based on multiple geometric feature learning;
- Random rescaling. The dental model is zoomed in or out randomly and appropriately;
- Randomly removal. Some triangular meshes are randomly removed from the dental models during training.

**Figure 2:**
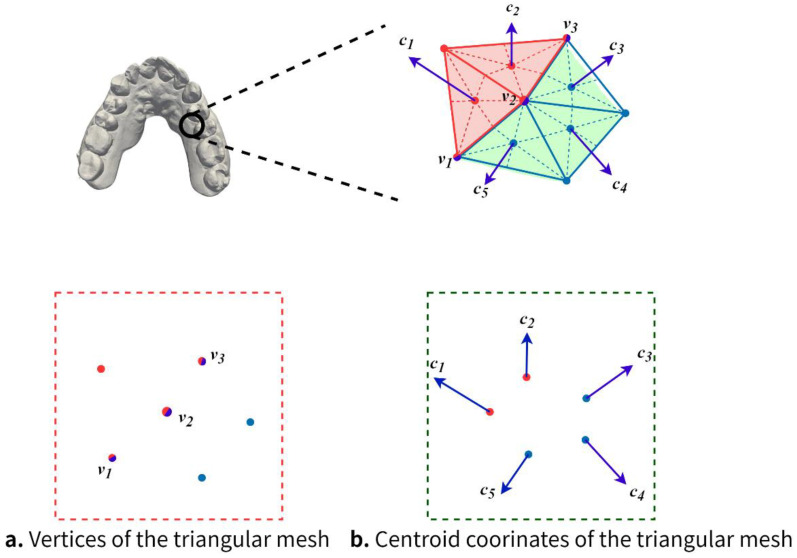
Mesh model point clouding segmentation.

In recent years, the point cloud segmentation methods represented by PointNet have achieved good segmentation results on point cloud models. However, there are many difficulties in applying these methods directly to the segmentation of the dental model. This is because the point cloud model is a three-dimensional model composed of discrete point sets, while the dental model used in this paper is a mesh model composed of many triangular meshes, which have different basic composition units and contain different geometric information. Since each triangular mesh contains vertex information, one possibility is to convert all the vertices of the mesh model into a point cloud model and then combine the point cloud segmentation method to complete the tooth segmentation. However, since the vertices will be shared by multiple triangular meshes, inferring the class of each triangular mesh by the class to which the vertices belong will generate ambiguity and inevitably lead to errors in tooth segmentation. As shown in Figure 2, two different colors represent two different segmentation categories, and the vertices $v_{1}$, $v_{2}$ and $v_{3}$ in Figure 2a are colored with two colors at the same time. The category they belong to creates ambiguity, and it is difficult to infer the category of each triangular mesh through these vertices. However, Figure 2b shows that the centroids $c_{1}$, $c_{2}$, $c_{3}$, $c_{4}$ and $c_{5}$ possess only one color since each triangular mesh contains only one centroid, and the class to which the centroid belongs is clear. Therefore, this paper uses the centroid of each triangular mesh as a guide for tooth segmentation, as shown in Equation (1):

(1) $$c_{i}=\left(\frac{x_{i}^{1}+x_{i}^{2}+x_{i}^{3}}{3},\frac{y_{i}^{1}+y_{i}^{2}+y_{i}^{3}}{3},\frac{z_{i}^{1}+z_{i}^{2}+z_{i}^{3}}{3}\right)$$

where $c_{i}$ denotes the coordinates of the centroid of triangular mesh i. $\left(x_{i}^{1},y_{i}^{1},z_{i}^{1}\right)$, $\left(x_{i}^{2},y_{i}^{2},z_{i}^{2}\right)$, and $\left(x_{i}^{3},y_{i}^{3},z_{i}^{3}\right)$ denote the three vertex coordinates of triangular mesh i. Unlike the vertices, each centroid has a one-to-one mapping relationship with each triangular mesh, and there is no case where a centroid is shared by multiple triangular meshes. Furthermore, the number of centroids is equal to the number of triangular meshes, while the number of vertices in the dental model is usually less than the number of triangular meshes, and a richer number of centroids is beneficial to improve the performance of neural networks [10,32].

### 3.2. Network Architecture Design

**Figure 3:**
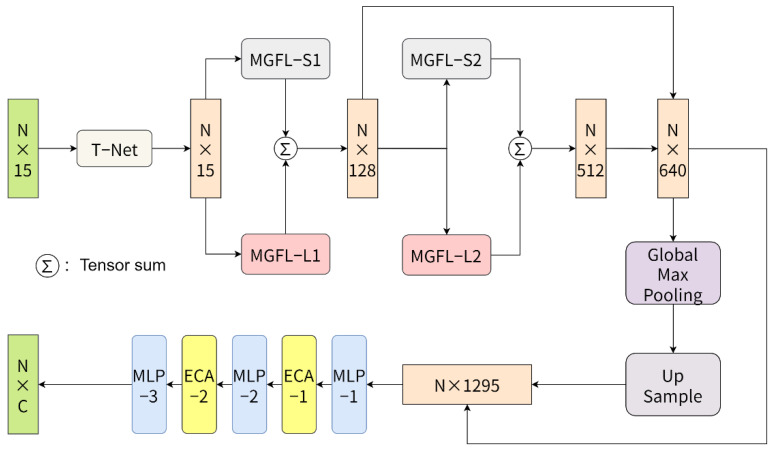
Overall network structure.

Figure 3 shows the overall network structure of our tooth segmentation algorithm. The network consists of the spatial transformation (T-Net) module [2], the multiple geometric feature learning module (MGFL), the multilayer perceptron (MLP), and the efficient channel attention (ECA). The input of the network is an N × 15 feature matrix extracted from the dental mesh model, where N is the number of triangular meshes of the dental model, and 15 is the feature dimension corresponding to each triangular mesh (containing the vertex coordinates, centroid coordinates, and normal vectors of the mesh). After T-Net, the feature alignment is completed. Then, the MGFLs with two different perceptual fields designed in this paper are fed, and the two outputs are continuously superimposed. The global feature information is then extracted from the final superimposed features by maximum pooling, followed by upsampling to restore the original feature dimensions. Finally, the features aligned by T-Net and the features before and after maximum pooling are fused, and the features are fed into MLP and ECA to complete feature-by-feature downsampling and channel optimization. The final output of the network is an N × C feature matrix, with each row representing the probability that each triangular mesh belongs to one of C (gums and 14 teeth) different classes.

#### 3.2.1. Multiple Geometric Feature Learning Module

**Figure 4:**
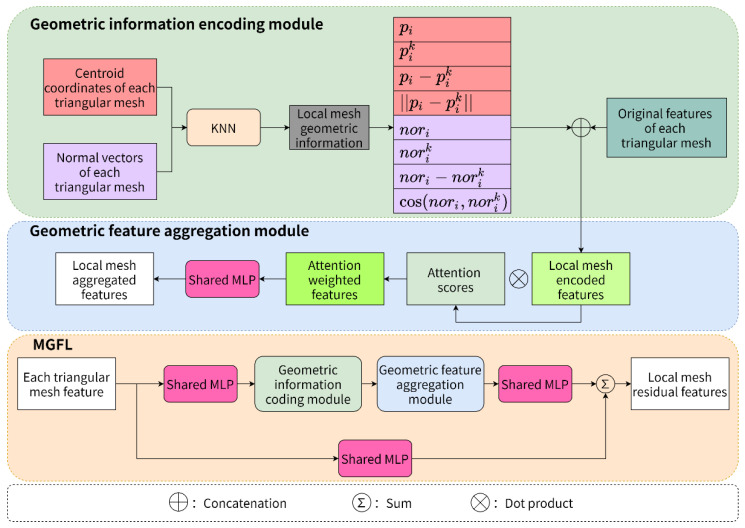
MGFL diagram. MGFL consists of shared MLP, geometric information encoding (GIE) module and geometric feature aggregation (GFA) module.

Considering that the normal vector is also important geometric information, combining it with vertex coordinate information can better represent the geometric features of the dental model. To further explore the contribution of geometric information with different attributes to the feature representation of the dental model, a multiple geometric feature learning (MGFL) module inspired by RandLA-Net is designed in this paper. As shown in Figure 4, MGFL consists of submodules, including the shared multilayer perceptron (shared MLP), geometric information encoding (GIE) module and geometric feature aggregation (GFA) module. These submodules are stacked together using skip connections to form a residual structure that can improve the network performance. The centroid coordinates, normal vectors and original features of each triangular mesh are used as the input data of MGFL to obtain semantically informative local mesh residual features.

The role of the geometric information encoding module is to encode the centroid coordinates and normal vectors of the meshes in the local region for enhancement and to capture the local mesh encoded features through spatial geometry information. Assume that the number of input triangle meshes is N and the number of neighbor meshes for each triangle mesh is K (including itself). First, for each mesh, use the K nearest neighbor search algorithm to compute K other meshes. For these K meshes, their centroid coordinates, normal vectors and other information are efficiently able to construct the local centroid coordinate feature set $p_{k}$ and local normal vector feature set $n_{k}$ of each mesh, as shown in Equations (2) and (3), respectively.

(2) $$p_{k}=p_{i}⊕p_{i}^{k}⊕\left(p_{i}-p_{i}^{k}\right)⊕\|p_{i}-p_{i}^{k}\|$$

(3) $$n_{k}=nor_{i}⊕nor_{i}^{k}⊕\left(nor_{i}-nor_{i}^{k}\right)⊕cos\left(nor_{i},nor_{i}^{k}\right)$$

(4) $$r_{i}^{k}=MLP\left(p_{k}⊕n_{k}\right)$$

where $p_{i}$ is the centroid coordinate of triangular mesh i, $p_{i}^{k}$ is the centroid coordinate of K other meshes of triangular mesh i, $p_{i}-p_{i}^{k}$ is the relative coordinate between triangular mesh i and other meshes, $∥p_{i}-p_{i}^{k}∥$ is the Euclidean distance between triangular mesh i and other meshes, $nor_{i}$ is the normal vector of triangular mesh i, $nor_{i}^{k}$ is the normal vector of K other meshes of triangular mesh i, $nor_{i}-nor_{i}^{k}$ is the normal vector difference between triangular mesh i and the other meshes, and $cos\left(nor_{i},nor_{i}^{k}\right)$ is the normal vector cosine similarity between triangular mesh i and the other meshes. Finally, as shown in Equation (4), the new triangular mesh geometric feature $r_{i}^{k}$ is obtained by cascading $p_{k}$ and $n_{k}$, and then $r_{i}^{k}$ is cascaded with the original features of each triangular mesh to obtain the local mesh encoded features and sent to the geometric feature aggregation module for processing.

In the feature extraction stage, it has become common practice for existing deep learning models to directly extract local features using average pooling or maximum pooling operations. However, such processing can easily lose useful feature information and affect the final performance. Therefore, to focus on the important features from the input features and avoid wastage of computational resources, the geometric feature aggregation module uses an attention mechanism to aggregate the local mesh encoded features. Specifically, for the augmented semantic feature set ${\hat{F}}_{i}=\left\{{\hat{f}}_{i}^{1}\cdots {\hat{f}}_{i}^{k}\cdots {\hat{f}}_{i}^{K}\right\}$ obtained from the geometric information encoding module, a function is designed to learn a unique attention score for each triangular mesh, which consists of a multilayer perceptron, as shown in Equation (5):

(5) $$s_{i}^{k}=g\left({\hat{f}}_{i}^{k},W\right)$$

where W is the learnable weight parameter of the multilayer perceptron, and $s_{i}^{k}$ is the learned attention score, which can automatically select more abstract geometric features and reduce or filter out the influence of other features. Finally, the attention weighted features are obtained by the dot product of the local mesh encoded features and the corresponding attention scores, as shown in Equation (6). Then the local mesh aggregation features are obtained by a multilayer perceptron.

(6) $${\tilde{f}}_{i}=\overset{K}{\underset{k=1}{\sum }}\left({\hat{f}}_{i}^{k}\cdot s_{i}^{k}\right)$$

#### 3.2.2. Global Feature Channel Optimization

The local geometric features of the dentition are obtained from the MGFL of different neighborhood perceptual fields in two different neighborhoods. Then the global geometric features of the dentition can be obtained by the global maximum pooling operation. The semantic information of each triangular mesh of the dentition can be comprehensively described by fusing the local and global geometric features together. However, the dimensionality of the fused features is very large. Hence in the final output stage of the neural networks, to quickly condense the decisive mesh semantic information and prevent the neural networks from being too complex, this paper achieves the goal of feature downscaling and channel optimization by stacking a small number of MLPs and efficient channel attention (ECA) [11].

**Figure 5:**
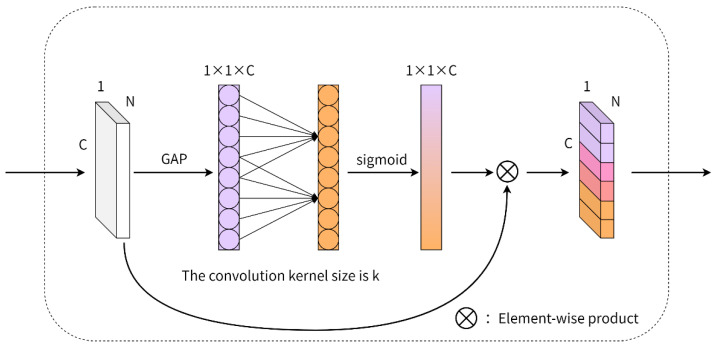
ECA diagram. N represents the number of input triangular meshes for the dental model, and C represents the feature dimension of each triangular mesh.

As shown in Figure 5, ECA-Net is a lightweight channel attention module that accomplishes cross-channel information exchange without reducing the dimensions through adaptive one-dimensional convolution. Specifically, each channel of the input feature is first pooled globally to generate the feature descriptor of each channel. Second, the cross-channel interaction range k is dynamically adjusted by the channel dimension C of the input feature, which represents that each channel has k nearest neighbor channels. Then, the local cross-channel interaction is completed by using one-dimensional convolution with convolution kernel size k, and the weight of each channel is obtained by using a sigmoid activation function. Finally, the weight of each corresponding channel of the original feature is weighted to complete the reoptimization of the original feature.

Compared with the fully connected layer, the one-dimensional convolution with kernel size k has fewer parameters and it only considers the interaction between its k nearest neighbor channels when interacting. This has higher computational efficiency and does not affect the overall complexity of the model. Generally, when the channel dimension C of the input features is large, appropriately increasing the k value will have better results. Thus, it is assumed that there is a positive relationship between the channel dimension C and k, which is expressed by a nonlinear function, as shown in Equation (7).

(7) $$C=2^{\left(\gamma *k-b\right)}$$

Using the deformable Formula (8)

(8) $$k={\left|t\right|}_{odd}={\left|\frac{\log_{2}\left(C\right)}{\gamma }+\frac{b}{\gamma }\right|}_{odd}$$

where ${\left|t\right|}_{odd}$ denotes the odd number closest to t. γ and b are fixed parameters, set to 2 and 1, respectively.

#### 3.2.3. More Network Details

**Figure 6:**
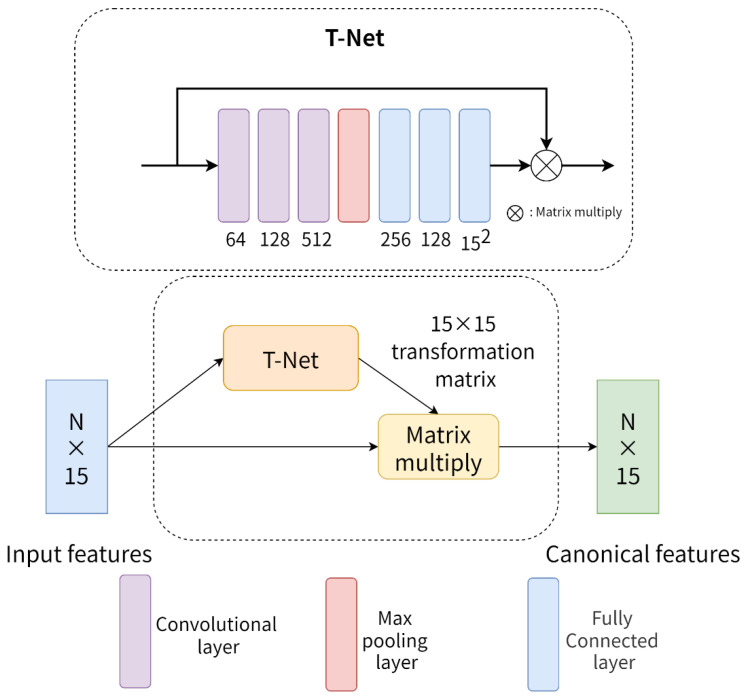
T-Net structure diagram.

The alignment of point cloud features using T-Net first appeared in PointNet [2]. As shown in Figure 6, T-Net consists of three convolutional layers, one maximum pooling layer and three fully connected layers. Each layer, except the maximum pooling layer and the last fully connected layer, is followed by batch normalization (BN) and a ReLU activation function. The module takes the original mesh features of the dental model as input. Then, firstly, the mesh features are gradually mapped to 64, 128 and 512 dimensions by three convolutional layers. Then, the global mesh features of size 1 × 512 are obtained by the maximum pooling layer. After this, the global mesh features are mapped to 256, 128 and 255 dimensions by three fully connected layers. Finally, the transformation matrix of size 15 × 15 is obtained by tensor shaping. This matrix is multiplied by the original mesh features, thus achieving a certain degree of mesh feature alignment and allowing the neural network to better learn the features of the dental mesh model.

As shown in Figure 3, for MGFL and MGFL-L, two variants of MGFL with different perceptual fields, the K values of K-nearest neighbor search are set to 3 and 20, the feature dimension of MGFL-S1 and MGFL-L1 output is 128, and the feature dimension of MGFL-S2 and MGFL-L2 output is 512. To compensate for the differences in geometric features of the two different perceptual fields and to compensate for the differences and deficiencies in the geometric features of the two different perceptual fields, the outputs of each pair of MGFLs are summed to complement each other. In the global feature channel optimization stage, the feature output dimension of MLP-1 and ECA-1 is 256, the feature output dimension of MLP-2 and ECA-2 is 128, and the feature output dimension of MLP-3 is C. The above MLP layers except MLP-3 are connected with batch normalization and ReLU activation functions. Finally, the network is obtained after softmax function processing and tensor deformation, and the results are output.

## 4. Experiments

The hardware environment for the experiments in this paper is an AMD Ryzen 5 3600 @ 4.2 GHz, NVIDIA GeForce RTX 3060Ti, 32 GB RAM. The software environment is Windows 10 64-bit version, Python 3.8, PyTorch 1.9.0+cu111, and CUDA 11.3. The Generalized Dice Loss (GDL) function [33] is used as the objective function for tooth classification, and the number of training rounds is 200. In addition, the Adam optimizer is used to train the neural networks, with the batch size set to 4. The initial learning rate is set to 0.001 which dynamically decays by the adaptive adjustment learning rate method (Reduce LR On Plateau) with a retention rate of 0.5 for the dropout parameter of the fully connected layer, using the change in the validation set loss as a reference.

### 4.1. Evaluation Metrics

To verify the validity of the experimental results in this paper, the overall accuracy (OA) and mean intersection over union (mIoU) are used to evaluate the accuracy of tooth segmentation. Overall accuracy represents the ratio of the number of correctly predicted meshes to the number of all meshes, and the mean intersection over union ratio represents the ratio of intersection and union between predicted values and labeled data (ground truth, GT), as shown in Equation (9).

(9) $$\mathrm{mIoU}=\frac{1}{k+1}\overset{k}{\underset{i=0}{\sum }}\frac{p_{ii}}{\sum_{j=0}^{k}p_{ij}+\sum_{j=0}^{k}p_{ji}-p_{ii}}$$

where there are k+1 segmentation categories; $p_{ij}$ denotes the probability that prediction triangular mesh i belongs to category j; $p_{ii}$ denotes the number of correct samples; and $p_{ji}$ denotes the number of incorrect samples.

### 4.2. Results

Our algorithm is compared with representative 3D shape segmentation methods (PointNet, RandLA-Net) and 3D tooth segmentation methods (MeshSegNet). The comparison of tooth segmentation accuracy results is shown in Table 1. Compared with PointNet and RandLA-Net, the proposed algorithm improves the OA by 3.5% and 6.4% and the mIoU by 7.0% and 15.6%, respectively. Compared with MeshSegNet in the field of tooth segmentation, the algorithm in this paper improves OA and mIoU by 1.1% and 3.4%, respectively. This shows that our algorithm achieves excellent tooth segmentation accuracy results, and the dentist can obtain fairly accurate tooth segmentation results, which have important applications for the subsequent treatment planning of patients.

We also consider the issue of tooth segmentation efficiency. As shown in Table 2, under the same environment, it can be seen that the training time per round of MeshSegNet is approximately 929.97 s and the segmentation time per dental model to be processed is approximately 3.20 s. In contrast, the training time per round of Pointnet, RandLA-Net and our algorithm is within 90 s, and the segmentation time per dental model is within 0.5 s or less. The computational efficiency of the proposed algorithms is about 11 times and 7 times higher than that of MeshSegNet, which involves the creation and computation of two adjacency matrices, and the size of these matrices is relatively large. This results in the high computational complexity of the algorithm. In contrast, our algorithm utilizes the efficient MGFL instead of the complex adjacency matrix to achieve higher computational efficiency.

**Figure 7:**
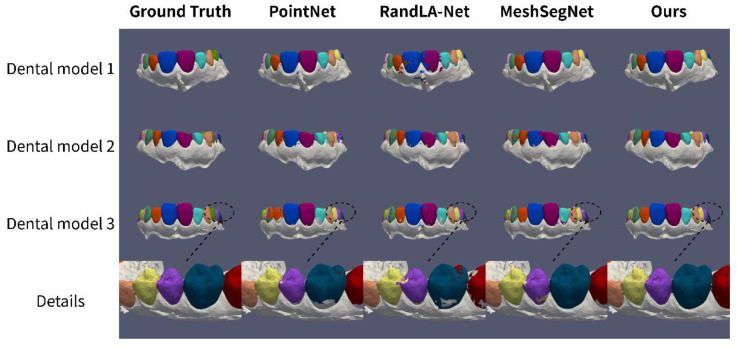
Comparison of tooth segmentation visualization results of different algorithms.

Three dental models were randomly selected from the test set, and the tooth segmentation results of PointNet, RandLA-Net, MeshSegNe, and our algorithm are visualized. The first three rows of Figure 7 represent the frontal views of the 1st, 2nd, and 3rd models, and the last row represents the lateral view of the 3rd model. From the first three rows of Figure 7, it can be seen that PointNet shows undersegmentation at the base of the crown of the incisors, oversegmentation in the gingiva around incisors, and more segmentation errors in adjacent incisors; RandLA-Net as a whole has a larger range of segmentation errors, with most incisors and cusps bearing category labels of other teeth and significant segmentation errors in the gingival part. This is mainly because PointNet and RandLA-Net focus more on point coordinate geometric information, which makes it difficult to use them directly in dental models with complex geometric information. MeshSegNet also has some degree of undersegmentation and adjacent tooth segmentation errors. The last row of Figure 7 further shows that MeshSegNet displays more obvious segmentation errors between adjacent molars and undersegmentation at the base of the crowns of the molars. This indicates that the feature extraction of MeshSegNet for teeth is relatively coarse because the complex geometric information of the dental model is simply used as a single feature vector. The tooth segmentation results of our algorithm are significantly better than those of PointNet and RandLA-Net and are more accurate than those of MeshSegNet in terms of details such as adjacent teeth and crown base. This shows that the proposed algorithm has a significant effect on the coding enhancement of different geometric features, thus improving the accuracy of tooth segmentation.

### 4.3. Ablation Study

#### 4.3.1. Effectiveness of Geometric Information Encoding Module

To evaluate the effectiveness of the geometric information encoding module, we implemented two deformation structures of the original network by changing the input form of the geometric information encoding module, including (1) the centroid coordinates of each triangular mesh (i.e., $p_{k}$) and (2) the normal vectors of each triangular mesh (i.e., $n_{k}$). These two deformation structures are compared with the original network design of our model, and the segmentation accuracy comparison results are shown in Table 3. We see that the network achieves higher segmentation accuracy when the input of the geometric information encoding module contains both $p_{k}$ and $n_{k}$. Specifically, for OA and mIoU, our algorithm improves by 2.4% and 5.7% over the former and 1.0% and 2.2% over the latter, respectively, compared to the deformation structure containing only $p_{k}$ or $n_{k}$. This indicates that encoding only the geometric information of a single attribute cannot fully extract the dental model features, and it is difficult to obtain better tooth segmentation results. It also shows that encoding the spatial coordinate information together with the normal vector helps to describe the mesh features of the dental model from a complementary perspective.

#### 4.3.2. Effectiveness of the Double Branch MGFL

To verify the effectiveness of the double branch MGFL, a series of experiments were designed. Specifically, the MGFL modules were split with two different sensory fields originally used in combination, including (1) MGFL with a large sensory field removed (i.e., MGFL-S) and (2) MGFL with a small sensory field removed (i.e., MGFL-L). These two deformation structures are compared with the original network design and the segmentation accuracy comparison results are shown in Table 4. It can be seen that the proposed algorithm’s accuracy is 2.2% and 5.6% higher than that of MGFL-S in OA and mIoU, respectively, and 0.8% and 1.2% higher than that of MGFL-L, respectively. This means that MGFLs with different receptive fields have different fine-grained granularities for local region feature extraction, and the combination can provide more comprehensive local geometric information for tooth segmentation.

#### 4.3.3. Effectiveness of Global Feature Channel Optimization

The fusion of local to global features has been shown to be effective for downstream tasks [2], and the fused features need to undergo feature dimensionality reduction to obtain the desired neural network output. The optimization of feature channels during the dimensionality reduction process can effectively utilize both local and global features of the dental model. To verify the effectiveness of ECA for feature channel optimization, a deformation structure of the original network is designed. Specifically, ECA is not used as a postprocessing step in the feature reduction part behind the original network. This deformation structure is compared with the original network design, and the segmentation accuracy comparison results are shown in Table 5. As shown, our algorithm is 0.5% and 1.6% more accurate than the deformation structure in OA and mIoU, respectively. Thus, it can be concluded that adding ECA as postprocessing in the feature downscaling part can achieve the purpose of channel optimization and obtain better tooth segmentation results.

#### 4.3.4. Effect of Different Input Triangular Mesh Numbers on Tooth Segmentation Results

In order to investigate the effects of different input triangular mesh numbers on the tooth segmentation results, the input triangular mesh numbers of the dental model were adjusted in this paper. Specifically, the number of input triangular meshes was assumed to be N. A total of five different sizes of N = 6000, N = 7000, N = 8000, N = 9000, and N = 10,000 were set as the input for network training. As shown in Table 6, the test results show that compared with N = 6000 and N = 7000, when the number of input triangular meshes is 8000–10,000, an improvement of about 2.0% and 2.6% is achieved in OA and mIoU, respectively. Moreover, as N gradually increases from 8000 to 10,000, the improvement in OA and mIoU gradually becomes smaller. This indicates that the accuracy of tooth segmentation is improved as N increases, while when N is small, the geometric features of the tooth learned by the network are more vestigial, and it is difficult to obtain good tooth segmentation results.

## 5. Conclusions

In this paper, we propose a tooth segmentation method based on multiple geometric feature learning for segmenting 3D dental mesh models. In this paper, a multiple geometric feature learning module is designed to enable the neural network to extract geometric features that are more discriminative across different meshes. When processing local-to-global fusion features, MLP and ECA are used to complete feature downscaling and channel optimization layer by layer to further improve the tooth segmentation results. The experimental results show that the algorithm in this paper achieves better results in terms of tooth segmentation accuracy and efficiency, and the overall process is automatic and efficient without complex and lengthy user interaction. However, since most of the dental models in the dataset are normal teeth data, in the future, we plan to introduce extreme dental models such as missing teeth as the training set to further improve the model’s generalization ability. In addition, considering that the annotation of 3D dental models is a time-consuming task, the weakly supervised point cloud segmentation method is a promising research direction, and in the future we may be able to obtain good tooth segmentation results using incompletely annotated dental models. Finally, collaboration with other areas of work may be beneficial to the depth of this study [34,35], and such collaboration has an important role to play in the development of each area.

## Figures and Tables

**Table 1:** Comparison of tooth segmentation accuracy of different algorithms.

Method	OA	mIoU
PointNet	95.9	89.3
RandLA-Net	92.2	81.4
MeshSegNet	97.3	92.9
Ours	98.4	96.3

**Table 2:** Comparison of tooth segmentation efficiency of different algorithms.

Method	Training (s/Epoch)	Prediction (s/Dental)
PointNet	20.83	0.24
RandLA-Net	56.15	0.34
MeshSegNet	929.97	3.20
Ours	86.55	0.48

**Table 3:** The segmentation result for our original network and two variants implemented by changing Geometric Information Encoding Module inputs.

Structure	OA	mIoU
$\mathrm{only}\;p_{k}$	96.0	90.6
$\mathrm{only}\;n_{k}$	97.4	94.1
both include	98.4	96.3

**Table 4:** The segmentation results by using single or double branch MGFL.

Structure	OA	mIoU
MGFL-S	96.2	90.7
MGFL-L	97.6	95.1
both include	98.4	96.3

**Table 5:** The comparison of segmentation results with or without ECA in the global feature channel optimization stage.

Structure	OA	mIoU
without ECA	97.9	94.7
with ECA	98.4	96.3

**Table 6:** The segmentation results of different input number of triangular meshes.

Input Number of Triangular Meshes	OA	mIoU
6000	87.7	80.5
7000	95.1	91.1
8000	97.1	93.7
9000	98.4	96.3
10,000	98.1	95.4

## Data Availability

Not applicable.
